# The New Anatomy Act

**Published:** 1883-04

**Authors:** 


### THE NEW ANATOMY ACT.

On the 1st instant there came into force the Provisional Act "to amend and consolidate the various Acts respecting the study of Anatomy." It is a new departure in the way of legislation for the prevention of body-snatching, and if faithfully carried out will, it is to be hoped, prove effectual. As it is very important that all members of the profession in this Province should be acquainted with the provisions of this Act, we shall draw attention to the various sections of which it is composed. The Province is first of all divided into two sections, the "Quebec Section" and the "Montreal Section," which shall comprise such judicial districts as may be specified by the Lieutenant-Governor. Then, the Lieutenant-Governor is to appoint an Inspector of Anatomy for each of these sections, and a Sub-Inspector of Anatomy for each judicial district except those of Quebec and

Montreal, at which places the Inspectors themselves act as such. The next section provides that every unclaimed body in institutions receiving Government aid shall be delivered, through the Inspectors, to the different schools of medicine. To prevent such action a claim for the corpse must be made inside of twenty-four hours of the person's death, by a relative within the degree of cousin-german. All superintendents or directors of institutions with public grants, and all coroners, are obliged to immediately notify the Inspector or Sub-Inspector of there being an unclaimed corpse under their control. Provision is made that the Inspectors keep registers of all such notifications received ; that they impartially distribute to the schools of medicine the corpses thus received, in proportion to the number of their students ; and that they inspect the dissecting rooms and order the decent interment of the remains. The Inspector is to receive $10 for each body so supplied, in addition to cost of transport and burial. A fine of from $100 to $200 is imposed upon the Superintendent of the above-named institutions who fails to notify as provided in the Act ; and a similar fine is laid upon any school of medicine which shall be found to have received and used for dissection any body not procured through the Inspector. The Inspector or Sub-Inspector is bound to appear within eight days before the *curé*, or clergyman, of the parish wherein has occurred the death of any unclaimed person, and to make an entry on the death-register of the date of death, etc., and of the manner of disposition of the body. All previous Acts are repealed.

We believe this law will work well, will provide an ample supply of dissecting material to all the schools, and at the same time put a stop to the disgraceful desecrations of graveyards and vaults, which have of late become so common. The principal defect in all previous Acts, and which we have had occasion several times to point out, has been the absence of any penal clause with reference to non-notification of the existence of unclaimed bodies in our public institutions. A very false feeling on this subject has long existed among the directors of several of these establishments; but we trust now that their attention is strongly directed toward the necessity of their performing this public duty, they will loyally comply with the terms of this enactment. It only remains for the Government to make judicious appointments of the persons to fill the important post of Inspector, and we hope that these officials will at once enter vigorously upon the performance of their public functions, and with impartiality—without fear, favor or affection—see to it that the intentions of the law are carried out in every institution in the Province which gets a grant from the public purse. On the other hand, let the schools of medicine accept cordially the legislation thus effected in their best interests; let them insist upon their rights as defined by the law, whilst at the same time refusing to countenance in any way the illicit traffic in bodies which has hitherto prevailed, only through the incompetency of officials and the refusal of responsible persons to furnish the assistance they will now be obliged to give. Let both parties concerned in this matter unite to put this Act fairly into operation, and body-snatching will soon be numbered among the lost arts.—*Canada Med. Journal.*

